# 2-(2-Methyl-5-nitro-1*H*-imidazol-1-yl)ethyl 3-bromo­benzoate

**DOI:** 10.1107/S1600536809015499

**Published:** 2009-04-30

**Authors:** Sher Bahadur, Itrat Anis, Muhammad Raza Shah, Kuldip Singh

**Affiliations:** a HEJ Research Institute of Chemistry, International Center for Chemical and Biological Sciences, University of Karachi, Karachi 75270, Pakistan; b Department of Chemistry, University of Karachi, Karachi 75270, Pakistan; c Department of Chemistry, University of Leicester, George Porter Building, University Road, Leicester LE1 7RH, England

## Abstract

The mol­ecule of the title compound, C_13_H_12_BrN_3_O_4_, is non-planar, as indicated in the dihedral angle of 59.5 (4)° formed between the least-squares planes through the imidazole and benzene rings. In the crystal, mol­ecules are connected *via* C—H⋯O contacts, forming a supra­molecular chain.

## Related literature

For potential pharmacological uses of benzoic acid derivatives, see: Correa-Basurto *et al.* (2005 ▶); Jetten *et al.* (1987 ▶); Kelly *et al.* (2007 ▶); Sato *et al.* (2005 ▶). For a related structure, see: Wang *et al.* (2008 ▶).
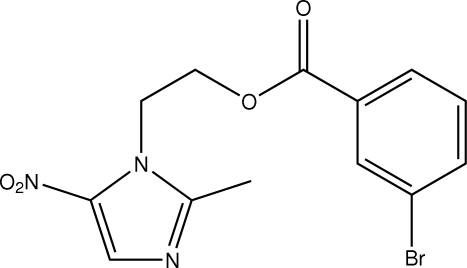


## Experimental

### 

#### Crystal data

C_13_H_12_BrN_3_O_4_
*M* *_r_* = 354.17
Monoclinic, 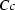

*a* = 11.871 (2) Å
*b* = 19.840 (4) Å
*c* = 7.1983 (13) Å
β = 124.488 (3)°
*V* = 1397.4 (4) Å^3^
*Z* = 4
Mo *K*α radiation
μ = 2.96 mm^−1^
*T* = 150 K
0.20 × 0.07 × 0.03 mm

#### Data collection

Bruker SMART APEX CCD area-detector diffractometer
Absorption correction: multi-scan (*SADABS*; Sheldrick, 1996 ▶) *T*_min_ = 0.589, *T*_max_ = 0.916
5456 measured reflections
2680 independent reflections
1636 reflections with *I* > 2σ(*I*)
*R*_int_ = 0.087

#### Refinement

*R*[*F* ^2^ > 2σ(*F* ^2^)] = 0.061
*wR*(*F* ^2^) = 0.119
*S* = 0.86
2680 reflections
191 parameters
2 restraints
H-atom parameters constrained
Δρ_max_ = 0.66 e Å^−3^
Δρ_min_ = −0.55 e Å^−3^
Absolute structure: Flack (1983 ▶), 1301 Friedel pairs
Flack parameter: 0.091 (17)

### 

Data collection: *SMART* (Bruker, 2001 ▶); cell refinement: *SAINT* (Bruker, 1999 ▶); data reduction: *SAINT*; program(s) used to solve structure: *SHELXS97* (Sheldrick, 2008 ▶); program(s) used to refine structure: *SHELXL97* (Sheldrick, 2008 ▶); molecular graphics: *SHELXTL* (Sheldrick, 2008 ▶); software used to prepare material for publication: *SHELXTL*.

## Supplementary Material

Crystal structure: contains datablocks I, global. DOI: 10.1107/S1600536809015499/tk2425sup1.cif

Structure factors: contains datablocks I. DOI: 10.1107/S1600536809015499/tk2425Isup2.hkl

Additional supplementary materials:  crystallographic information; 3D view; checkCIF report

## Figures and Tables

**Table 1:** Hydrogen-bond geometry (Å, °)

*D*—H⋯*A*	*D*—H	H⋯*A*	*D*⋯*A*	*D*—H⋯*A*
C4—H4a⋯O2^i^	0.99	2.59	3.494 (10)	152

Symmetry code: (i) 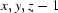.

**Fig. 1.**
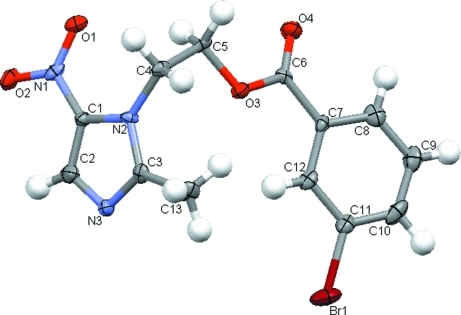
Molecular Structure of (I) show atom labelling and 30% displacement ellipsoids.

## supplementary crystallographic information

### Comment

Derivatives of benzoic acid offer promise as compounds that possess multifunctional physiological activity (hypolesterolemic, antitumor, antithrombic, *etc*.) and do not cause hypervitaminosis and other side-effects (Jetten *et al.* 1987). It has been reported that synthesized benzoic acid derivatives of the amide- and chalcone-series show inhibitory activity of squamous cell differentiation of rabbit traeheal epithelial cell but induce differentiation of mouse embryonal carcinoma F9 and human promyelocytic leukemia HL60 cells (Correa-Basurto *et al.*, 2005). *p*-Aminobenzoic acid derivatives were evaluated as acetylcholinesterase inhibitors (AChEIs) (Sato *et al.*, 2005). Benzoic acid derivatives have also been found to exhibit cytotoxic effects on the MDA-MB-435-S—F breast cancer cell line (Kelly *et al.*, 2007).

In the crystal structure of the title compound (I), Fig. 1, the key C=O and C—N bond distances are in agreement with those observed in the related structure of imidazolmethyl phthalimide (Wang *et al.*, 2008).

### Experimental

Metronidazole (5 g, 29.23 mmol) was added to 3-bromobenzoic acid (7.64 g, 38.01 mmol) dissolved in anhydrous CH_2_Cl_2_ (10 ml). Then 4-dimethylaminopyridine (0.15 equiv.) and dicyclohexylcarbodiimide (1.25 equiv) were added, and the resulting solution stirred. After 12 h, the solvent was evaporated under reduced pressure. The crude reaction mixture was subjected to flash column chromatography over silica gel, successively eluting with n-hexane–ethyl acetate (3:7) which afforded (I) in 70% yield. Colorless crystals were obtained from the slow evaporation of a CH_2_Cl_2_ solution of (I).

### Refinement

H atoms were placed in calculated positions, C—H = 0.95–0.99 Å, and included in the riding model approximation with U_iso_ set to 1.5U_eq_(C) for methyl-H atoms and 1.2U_eq_(C) for remaining H atoms.

### Crystal data

**Table:**

C_13_H_12_BrN_3_O_4_	*F*(000) = 712
*M**_r_* = 354.17	*D*_x_ = 1.683 Mg m^−^^3^
Monoclinic, *C**c*	Mo *K*α radiation, λ = 0.71073 Å
Hall symbol: C -2yc	Cell parameters from 616 reflections
*a* = 11.871 (2) Å	θ = 3.7–23.3°
*b* = 19.840 (4) Å	µ = 2.96 mm^−^^1^
*c* = 7.1983 (13) Å	*T* = 150 K
β = 124.488 (3)°	Needle, colourless
*V* = 1397.4 (4) Å^3^	0.20 × 0.07 × 0.03 mm
*Z* = 4	

### Data collection

**Table:**

Bruker APEX 2000 CCD area-detector diffractometer	2680 independent reflections
Radiation source: fine-focus sealed tube	1636 reflections with *I* > 2σ(*I*)
graphite	*R*_int_ = 0.087
φ and ω scans	θ_max_ = 26.0°, θ_min_ = 2.1°
Absorption correction: multi-scan (*SADABS*; Sheldrick, 1996)	*h* = −14→14
*T*_min_ = 0.589, *T*_max_ = 0.916	*k* = −24→23
5456 measured reflections	*l* = −8→8

### Refinement

**Table:**

Refinement on *F*^2^	Secondary atom site location: difference Fourier map
Least-squares matrix: full	Hydrogen site location: inferred from neighbouring sites
*R*[*F*^2^ > 2σ(*F*^2^)] = 0.061	H-atom parameters constrained
*wR*(*F*^2^) = 0.119	*w* = 1/[σ^2^(*F*_o_^2^) + (0.0345*P*)^2^] where *P* = (*F*_o_^2^ + 2*F*_c_^2^)/3
*S* = 0.86	(Δ/σ)_max_ = 0.001
2680 reflections	Δρ_max_ = 0.66 e Å^−^^3^
191 parameters	Δρ_min_ = −0.55 e Å^−^^3^
2 restraints	Absolute structure: Flack (1983), 1301 Friedel pairs
Primary atom site location: structure-invariant direct methods	Flack parameter: 0.091 (17)

### Special details

**Table:**

Geometry. All e.s.d.'s (except the e.s.d. in the dihedral angle between two l.s. planes) are estimated using the full covariance matrix. The cell e.s.d.'s are taken into account individually in the estimation of e.s.d.'s in distances, angles and torsion angles; correlations between e.s.d.'s in cell parameters are only used when they are defined by crystal symmetry. An approximate (isotropic) treatment of cell e.s.d.'s is used for estimating e.s.d.'s involving l.s. planes.
Refinement. Refinement of *F*^2^ against ALL reflections. The weighted *R*-factor *wR* and goodness of fit *S* are based on *F*^2^, conventional *R*-factors *R* are based on *F*, with *F* set to zero for negative *F*^2^. The threshold expression of *F*^2^ > σ(*F*^2^) is used only for calculating *R*-factors(gt) *etc*. and is not relevant to the choice of reflections for refinement. *R*-factors based on *F*^2^ are statistically about twice as large as those based on *F*, and *R*- factors based on ALL data will be even larger.

### Fractional atomic coordinates and isotropic or equivalent isotropic displacement parameters (Å^2^)

**Table:**

	*x*	*y*	*z*	*U*_iso_*/*U*_eq_	
Br1	0.81001 (10)	0.59782 (5)	0.72927 (13)	0.0588 (3)	
O1	0.0138 (5)	0.6343 (3)	0.5323 (9)	0.0363 (14)	
O2	0.0896 (5)	0.7074 (3)	0.8019 (9)	0.0434 (15)	
O3	0.2933 (5)	0.5475 (3)	0.4184 (9)	0.0309 (14)	
O4	0.2623 (6)	0.4375 (3)	0.4381 (11)	0.0437 (16)	
N1	0.0966 (6)	0.6786 (3)	0.6597 (11)	0.0322 (16)	
N2	0.2152 (6)	0.6769 (3)	0.4669 (9)	0.0234 (14)	
N3	0.3904 (6)	0.7449 (3)	0.6962 (11)	0.0324 (16)	
C1	0.2023 (8)	0.6976 (4)	0.6362 (12)	0.0261 (17)	
C2	0.3074 (10)	0.7391 (4)	0.7650 (18)	0.042 (3)	
H2	0.3216	0.7622	0.8924	0.050*	
C3	0.3307 (8)	0.7090 (4)	0.5099 (13)	0.0281 (18)	
C4	0.1261 (7)	0.6329 (4)	0.2730 (12)	0.0280 (18)	
H4A	0.1413	0.6422	0.1537	0.034*	
H4B	0.0295	0.6436	0.2115	0.034*	
C5	0.1513 (7)	0.5597 (4)	0.3332 (13)	0.0294 (19)	
H5A	0.1344	0.5490	0.4498	0.035*	
H5B	0.0903	0.5315	0.1988	0.035*	
C6	0.3328 (8)	0.4832 (4)	0.4592 (12)	0.0222 (18)	
C7	0.4765 (8)	0.4759 (4)	0.5349 (12)	0.0263 (19)	
C8	0.5272 (9)	0.4134 (4)	0.5409 (13)	0.041 (2)	
H8	0.4686	0.3754	0.4966	0.049*	
C9	0.6614 (10)	0.4036 (5)	0.6098 (14)	0.048 (2)	
H9	0.6962	0.3596	0.6198	0.057*	
C10	0.7422 (9)	0.4597 (5)	0.6630 (13)	0.049 (3)	
H10	0.8333	0.4538	0.7053	0.059*	
C11	0.6978 (8)	0.5231 (4)	0.6579 (12)	0.035 (2)	
C12	0.5625 (7)	0.5325 (4)	0.5850 (12)	0.032 (2)	
H12	0.5277	0.5768	0.5688	0.038*	
C13	0.3796 (8)	0.7029 (4)	0.3597 (13)	0.043 (2)	
H13A	0.3319	0.7359	0.2373	0.064*	
H13B	0.3608	0.6573	0.2962	0.064*	
H13C	0.4781	0.7115	0.4472	0.064*	

### Atomic displacement parameters (Å^2^)

**Table:**

	*U*^11^	*U*^22^	*U*^33^	*U*^12^	*U*^13^	*U*^23^
Br1	0.0321 (4)	0.0955 (8)	0.0384 (5)	−0.0218 (7)	0.0137 (4)	0.0017 (7)
O1	0.025 (3)	0.046 (4)	0.035 (3)	−0.002 (3)	0.014 (3)	0.006 (3)
O2	0.038 (3)	0.065 (4)	0.039 (3)	0.001 (3)	0.028 (3)	−0.007 (3)
O3	0.025 (3)	0.031 (3)	0.036 (4)	0.002 (3)	0.017 (3)	0.005 (3)
O4	0.042 (4)	0.031 (3)	0.062 (4)	−0.003 (3)	0.032 (4)	0.005 (3)
N1	0.021 (4)	0.044 (4)	0.030 (4)	0.007 (3)	0.014 (3)	0.013 (3)
N2	0.025 (4)	0.028 (4)	0.018 (3)	−0.009 (3)	0.012 (3)	−0.003 (3)
N3	0.031 (4)	0.036 (4)	0.036 (4)	−0.007 (3)	0.022 (4)	−0.012 (3)
C1	0.024 (4)	0.031 (4)	0.025 (4)	−0.005 (4)	0.015 (4)	−0.006 (4)
C2	0.030 (5)	0.030 (5)	0.059 (7)	0.003 (5)	0.021 (5)	0.000 (5)
C3	0.035 (5)	0.024 (4)	0.030 (5)	0.006 (4)	0.021 (4)	0.008 (4)
C4	0.027 (4)	0.039 (5)	0.022 (4)	−0.005 (4)	0.017 (4)	0.000 (4)
C5	0.021 (4)	0.043 (5)	0.028 (5)	−0.003 (4)	0.017 (4)	0.008 (4)
C6	0.027 (5)	0.028 (5)	0.010 (4)	−0.004 (4)	0.010 (3)	−0.002 (4)
C7	0.024 (4)	0.037 (5)	0.019 (4)	0.005 (4)	0.013 (4)	−0.002 (4)
C8	0.044 (6)	0.046 (6)	0.024 (5)	0.004 (5)	0.014 (4)	0.003 (4)
C9	0.049 (6)	0.049 (6)	0.041 (6)	0.022 (5)	0.023 (5)	0.011 (5)
C10	0.038 (5)	0.085 (8)	0.035 (6)	0.012 (5)	0.027 (5)	0.012 (5)
C11	0.029 (5)	0.050 (6)	0.021 (4)	0.005 (4)	0.011 (4)	0.000 (4)
C12	0.025 (4)	0.045 (5)	0.021 (4)	0.009 (4)	0.010 (4)	0.009 (4)
C13	0.053 (6)	0.043 (5)	0.044 (5)	−0.010 (5)	0.035 (5)	0.006 (4)

### Geometric parameters (Å, °)

**Table:**

Br1—C11	1.861 (8)	C4—H4B	0.9900
O1—N1	1.248 (7)	C5—H5A	0.9900
O2—N1	1.216 (8)	C5—H5B	0.9900
O3—C6	1.334 (9)	C6—C7	1.475 (10)
O3—C5	1.451 (8)	C7—C8	1.369 (10)
O4—C6	1.183 (9)	C7—C12	1.420 (11)
N1—C1	1.410 (9)	C8—C9	1.389 (12)
N2—C1	1.374 (9)	C8—H8	0.9500
N2—C3	1.378 (9)	C9—C10	1.375 (11)
N2—C4	1.468 (8)	C9—H9	0.9500
N3—C3	1.317 (9)	C10—C11	1.356 (11)
N3—C2	1.334 (11)	C10—H10	0.9500
C1—C2	1.336 (12)	C11—C12	1.391 (10)
C2—H2	0.9500	C12—H12	0.9500
C3—C13	1.493 (10)	C13—H13A	0.9800
C4—C5	1.497 (10)	C13—H13B	0.9800
C4—H4A	0.9900	C13—H13C	0.9800
			
C6—O3—C5	115.6 (6)	H5A—C5—H5B	108.7
O2—N1—O1	123.3 (6)	O4—C6—O3	124.8 (7)
O2—N1—C1	117.7 (7)	O4—C6—C7	124.0 (8)
O1—N1—C1	119.0 (6)	O3—C6—C7	111.3 (7)
C1—N2—C3	105.0 (6)	C8—C7—C12	118.0 (7)
C1—N2—C4	129.7 (6)	C8—C7—C6	119.7 (8)
C3—N2—C4	125.2 (6)	C12—C7—C6	122.1 (7)
C3—N3—C2	104.3 (7)	C7—C8—C9	122.2 (8)
C2—C1—N2	105.7 (7)	C7—C8—H8	118.9
C2—C1—N1	128.8 (8)	C9—C8—H8	118.9
N2—C1—N1	125.4 (6)	C10—C9—C8	117.7 (8)
N3—C2—C1	113.0 (9)	C10—C9—H9	121.1
N3—C2—H2	123.5	C8—C9—H9	121.1
C1—C2—H2	123.5	C11—C10—C9	122.9 (8)
N3—C3—N2	111.8 (6)	C11—C10—H10	118.5
N3—C3—C13	125.1 (7)	C9—C10—H10	118.5
N2—C3—C13	123.0 (7)	C10—C11—C12	118.9 (8)
N2—C4—C5	112.5 (6)	C10—C11—Br1	121.6 (7)
N2—C4—H4A	109.1	C12—C11—Br1	119.3 (6)
C5—C4—H4A	109.1	C11—C12—C7	120.0 (8)
N2—C4—H4B	109.1	C11—C12—H12	120.0
C5—C4—H4B	109.1	C7—C12—H12	120.0
H4A—C4—H4B	107.8	C3—C13—H13A	109.5
O3—C5—C4	106.1 (6)	C3—C13—H13B	109.5
O3—C5—H5A	110.5	H13A—C13—H13B	109.5
C4—C5—H5A	110.5	C3—C13—H13C	109.5
O3—C5—H5B	110.5	H13A—C13—H13C	109.5
C4—C5—H5B	110.5	H13B—C13—H13C	109.5
			
C3—N2—C1—C2	−0.1 (8)	C6—O3—C5—C4	−173.1 (6)
C4—N2—C1—C2	−177.6 (7)	N2—C4—C5—O3	−59.7 (7)
C3—N2—C1—N1	178.8 (7)	C5—O3—C6—O4	−2.7 (11)
C4—N2—C1—N1	1.3 (12)	C5—O3—C6—C7	177.4 (6)
O2—N1—C1—C2	7.5 (12)	O4—C6—C7—C8	13.3 (12)
O1—N1—C1—C2	−173.8 (8)	O3—C6—C7—C8	−166.7 (7)
O2—N1—C1—N2	−171.2 (7)	O4—C6—C7—C12	−172.0 (7)
O1—N1—C1—N2	7.5 (10)	O3—C6—C7—C12	8.0 (10)
C3—N3—C2—C1	3.8 (10)	C12—C7—C8—C9	4.8 (12)
N2—C1—C2—N3	−2.3 (10)	C6—C7—C8—C9	179.8 (7)
N1—C1—C2—N3	178.8 (7)	C7—C8—C9—C10	−3.0 (13)
C2—N3—C3—N2	−3.8 (9)	C8—C9—C10—C11	2.2 (13)
C2—N3—C3—C13	176.0 (8)	C9—C10—C11—C12	−3.3 (13)
C1—N2—C3—N3	2.5 (8)	C9—C10—C11—Br1	−179.6 (6)
C4—N2—C3—N3	−179.8 (6)	C10—C11—C12—C7	5.1 (11)
C1—N2—C3—C13	−177.3 (7)	Br1—C11—C12—C7	−178.5 (6)
C4—N2—C3—C13	0.3 (11)	C8—C7—C12—C11	−5.8 (11)
C1—N2—C4—C5	−81.4 (9)	C6—C7—C12—C11	179.4 (7)
C3—N2—C4—C5	101.6 (8)		

### Hydrogen-bond geometry (Å, °)

**Table:**

*D*—H···*A*	*D*—H	H···*A*	*D*···*A*	*D*—H···*A*
C4—H4a···O2^i^	0.99	2.59	3.494 (10)	152

Symmetry codes: (i) *x*, *y*, *z*−1.
